# Estriol attenuates visceral adiposity and pulmonary artery smooth muscle cell proliferation via ERα-mediated signalling

**DOI:** 10.1093/ehjopen/oeag001

**Published:** 2026-01-20

**Authors:** Smriti Sharma, Joshua P Dignam, Gregor Aitchison, Rosemary Gaw, Ioannis Stasinopoulos, Ayman Gebril, Martin Wabitsch, Ruth Andrew, Margaret R MacLean

**Affiliations:** Strathclyde Institute of Pharmacy and Biomedical Sciences, University of Strathclyde, 161 Cathedral Street, G4 0RE Glasgow, UK; Department of Internal Medicine II, Division of Cardiology, Medical University of Vienna, Waehringer Guertel 18-20, Vienna A-1090, Austria; Strathclyde Institute of Pharmacy and Biomedical Sciences, University of Strathclyde, 161 Cathedral Street, G4 0RE Glasgow, UK; Centre for Microvascular Research, William Harvey Research Institute, Barts and The London School of Medicine and Dentistry, Queen Mary University of London, Charterhouse Square, London EC1M 6BQ, UK; Strathclyde Institute of Pharmacy and Biomedical Sciences, University of Strathclyde, 161 Cathedral Street, G4 0RE Glasgow, UK; Strathclyde Institute of Pharmacy and Biomedical Sciences, University of Strathclyde, 161 Cathedral Street, G4 0RE Glasgow, UK; Center for Cardiovascular Science, Queen's Medical Research Institute, University of Edinburgh, 47 Little France Crescent, Edinburgh EH16 4TJ, UK; Strathclyde Institute of Pharmacy and Biomedical Sciences, University of Strathclyde, 161 Cathedral Street, G4 0RE Glasgow, UK; Division of Pediatric Endocrinology and Diabetes, Department of Paediatrics and Adolescent Medicine, Ulm University Medical Center, Eythstrasse 24, 89075 Ulm, Germany; German Center for Child and Adolescent Health (DZKJ), partner site Ulm, 89075 Ulm, Germany; Center for Cardiovascular Science, Queen's Medical Research Institute, University of Edinburgh, 47 Little France Crescent, Edinburgh EH16 4TJ, UK; Strathclyde Institute of Pharmacy and Biomedical Sciences, University of Strathclyde, 161 Cathedral Street, G4 0RE Glasgow, UK

**Keywords:** Estriol, Obesity, Adipose tissue, Inflammation, Vascular remodeling

## Abstract

**Aims:**

Estriol (E3) is a natural estrogen produced during pregnancy whose physiological role in the adult cardiovascular and pulmonary systems remains poorly understood. Given the established association between estrogens and obesity, our study aims to investigate the interplay between obesity, E3, and their potential cardiopulmonary effects.

**Methods and results:**

Effect of E3 on the cardiopulmonary system was evaluated in lean and high-fat diet-induced obese mice using right heart catheterization. Plasma triglyceride and adipokines were quantified using immunological assays, and circulating E3 levels were measured via LC-MS/MS. *In vitro* experiments were carried out in a human adipocyte cell line and pulmonary artery smooth muscle cells (PASMCs) isolated from rats and patients with pulmonary arterial hypertension. E3 reduces visceral adipose tissue mass *in vivo*, primarily by attenuating adipocyte inflammation and proliferation. E3 treatment significantly reduced plasma leptin levels, contributing to improved metabolic profiles. In adipocytes, E3 reduced pro-proliferation and inflammatory markers while increasing the expression of antioxidant genes. Additionally, E3 reduced proliferation in isolated PASMCs and E3-induced signalling was observed to be mediated through the ERα receptors.

**Conclusion:**

Our findings demonstrate, for the first time, that E3 reduces visceral adipose tissue mass, indicating its role in modulating adipose tissue characteristics while concurrently enhancing metabolic profiles. These results lay the groundwork for future research to investigate the role of E3 in disease prevention and its therapeutic application in cardiopulmonary disorders.

## Graphical Abstract

**Graphical Abstract:**
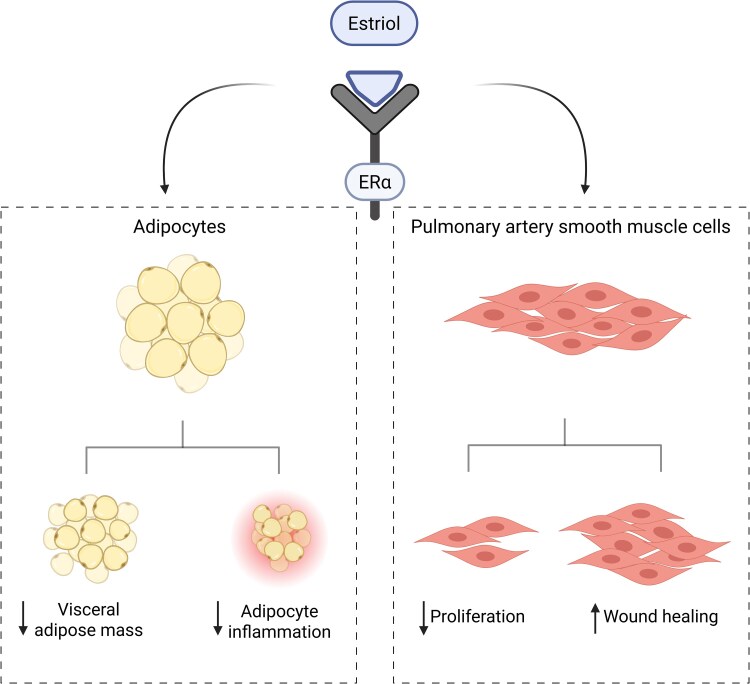

Translational Perspective Estriol (E3), a naturally occurring estrogen, may hold therapeutic potential for managing obesity and related cardiopulmonary conditions. E3 reduces visceral adipose tissue mass by modulating adipocyte inflammation and proliferation, improving metabolic profiles, and lowering plasma leptin levels. Additionally, E3’s effects on pulmonary artery smooth muscle cells (PASMCs) and its ability to reduce cell proliferation through ERα receptor signalling highlights its potential in treating pulmonary vascular diseases. These results provide a foundation for further clinical investigations into E3’s role in disease prevention and as a therapeutic option for obesity-related cardiopulmonary disorders.

## Introduction

Estriol (3,16,17 trihydroxy 13,5-oestriene, E3, or 16α-hydroxyestradiol [16OHE2]) is one of the three major endogenous estrogens alongside estrone (3-Hydroxyestra-13,5(10)-trien-17-one, E1) and estradiol (17β-Estradiol, E2). E3 is a relatively weak estrogen mainly produced by the fetoplacental unit^1^ and serves as an indicator of fetal health.^2^ It is believed to regulate uteroplacental blood flow and placental vascularisation during pregnancy.^3^ In non-pregnant pre-menopausal women, E3 is derived mainly from 16α-hydroxylation of E1 and E2 by liver cytochrome P450 (CYP) enzymes^4^ with urinary E3 concentrations ranging from 0.02 to 0.1 mg/day. However, in near-term pregnant women, these levels increase to 50–150 mg/day. Despite these high levels, circulating levels of E3 are similar to those of other estrogens due to its rapid metabolism and excretion. Of all three estrogens, E3 has the weakest binding affinity for the two nuclear estrogen receptors (ER), ERα and ERβ,^5^ but shows greater affinity for ERβ.^6^ Its reduced signalling through ERα is linked to a lower risk of uterine and breast cancer^7,8^ making it safe for long-term use. E3 has been used in hormone replacement therapy to improve bone density without affecting lipid levels, liver function, breast tissue or blood pressure.^9^

Obesity is a well-established contributor to cardiovascular disease (CVD), promoting endothelial dysfunction, microvascular remodelling, and cardiomyocyte toxicity, which collectively facilitate the development of atherosclerosis, coronary artery disease, cardiomyopathy, and heart failure. In parallel, excess adipose tissue exacerbates major CVD risk factors, including hypertension, type 2 diabetes, dyslipidemia, and chronic kidney disease.^10^ Notably, adipose tissue is also a well-known site of E2 production, especially in men and postmenopausal women.^11^ With respect to the role of estrogen and estrogen metabolites in cardiac physiology, our previous work has shown that 16OHE1 induces pulmonary hypertension in mice^12^ and that elevated urinary levels of 16OHE1 are associated with obesity-induced pulmonary vascular remodelling.^13^ We and others have also reported increased plasma levels of E2 and its metabolites—16OHE1 and E3 (16OHE2)—in patients with pulmonary arterial hypertension (PAH).^14–16^ Despite its presence in the adult circulation, the physiological and pathophysiological roles of E3 within the cardiovascular and pulmonary-vascular systems remain largely undefined. In this study, we have used PAH phenotype and associated features as a model disease to investigate the effects of E3, both *in vitro* and *in vivo*. Dysfunctional bone morphogenetic protein receptor type II (BMPR2) signalling is central to PAH pathogenesis, promoting aberrant proliferation of pulmonary vascular cells and perivascular inflammation.^17^ Given that estrogens have been reported to modulate BMPR2 expression and activity,^18^ we examined BMPR2-related signalling to explore its potential contribution to the E3-mediated effects.

## Methods

### Animal studies

All procedures followed the Animals (Scientific Procedures) Act 1986, ARRIVE guidelines, and National Institutes of Health guidelines for animal care (NIH publication No.85–23, revised 1996). Both male and female mice were included in the study to assess potential sex-specific differences in response to E3, particularly given the low baseline E3 levels in males. Moreover, it has been shown that the effects of E3 are not sex-specific.^19^ Male and female C57BL/6 mice (21–22 weeks old) were injected intraperitoneally with vehicle (4% ethanol) or E3 (1.5 mg/kg/day based on previous work with 16OHE1^12^) for 14 days before hemodynamic assessment. In the high-fat diet (HFD) study, 8–12-week-old mice were fed a standard diet (STD) or HFD containing 42% energy from fat, 43% from carbohydrate and 15% from protein (Western rodent diet, Special Diet Services, UK) for 24 weeks. After 22 weeks on HFD, mice received daily vehicle or E3 injections and were weighed weekly. All animals were group-housed under a 12-hour light/dark cycle with *ad libitum* access to food and water. Female mice were housed together to promote synchronisation of estrous cycles.

### Hemodynamic measurement and tissue collection

Right ventricular systolic pressure (RVSP) and heart rate were measured by right heart catheterization through the right jugular vein using a PVR-1045 Millar pressure–conductance catheter (Millar Instruments, Houston, TX) under terminal anaesthesia as described before.^20^ Blood was drawn by apical puncture, and the heart was excised. Atria were removed, and the right ventricle (RV) was separated from the left ventricle (LV) and septum (S), weighed and processed for further analysis. Right ventricular hypertrophy (RVH) was assessed by measuring the Fulton Index (RV weight/LV+S weight) relative to tibia length. Analysis was carried out in a blinded fashion.

### White adipose tissue harvest

Visceral white adipose tissue (vWAT; comprising perirenal and perigonadal fat) was collected, weighed and normalized to tibia length. The adrenal gland was carefully removed from the vWAT due to its high steroid content. Harvested adipose tissue was briefly washed in cold PBS and snap frozen until further use.

### Liquid chromatography with tandem mass spectrometry analysis

Mouse plasma samples were subjected to liquid chromatography with tandem mass spectrometry (LC-MS/MS) analysis using the method of Denver et al^21^ with slight modifications. Detailed methodology is provided in the supplemental methods.

### Human Simpson-Golabi-Behmel syndrome cells

Human Simpson-Golabi-Behmel syndrome (SGBS) preadipocyte cells were kindly provided by Prof. Martin Wabitsch (Ulm University Medical Center, Germany). Detailed methodology is provided in the supplemental methods.

### Statistical analysis

Normal distribution of the data was confirmed using the Shapiro-Wilk test. For comparison between two groups, a two-tailed, unpaired Student’s *t* test or non-parametric Mann–Whitney U test was used. For multi-group comparisons, one-way or two-way ANOVA followed by a Šídák multiple-comparisons test was used with adjustment for multiplicity. Statistical analysis was carried out in GraphPad Prism (v10; GraphPad software). *P* < 0.05 was considered statistically significant, and data are represented as mean ± SEM.

Detailed methodology for histology, immunoassay, immunoblotting, triglyceride assay, and *in-vitro* experiments using rat and human PASMCs is provided in the supplemental methods.

## Results

### E3 administration decreases pro-fibrotic gene expression in mouse lungs and RV

**Figure 1:**
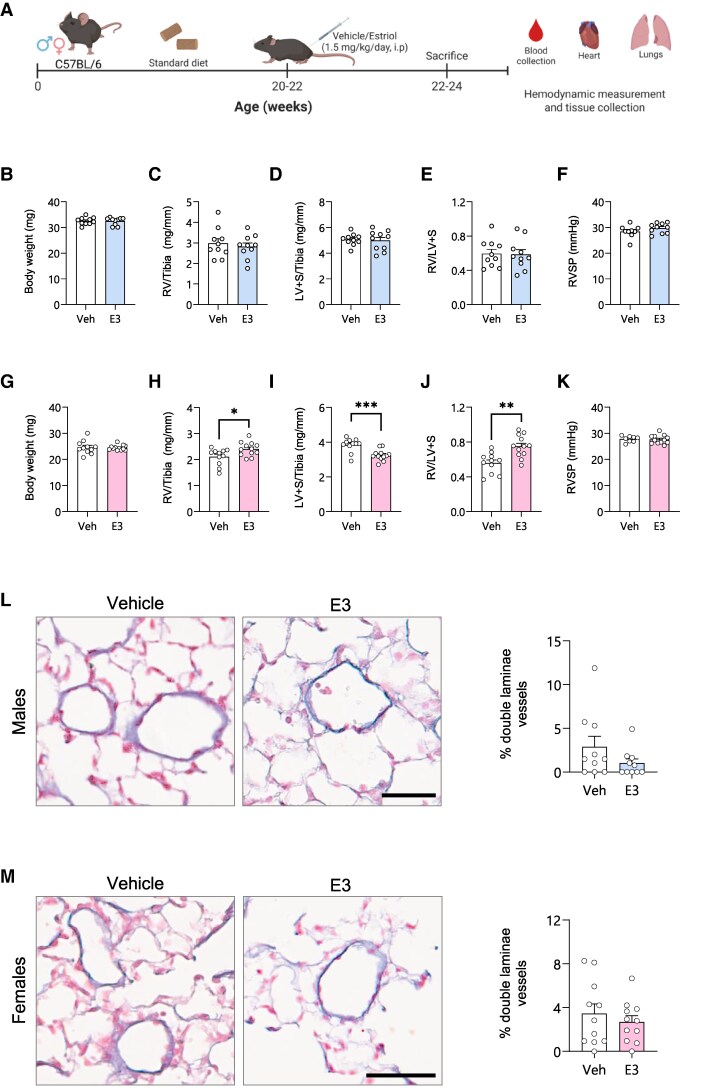
Effect of estriol (E3) on cardiac physiology and hemodynamics in C57BL/6 mice. Schematic representation of the animal experiments (*A*). Absolute body weight (*B*, *G*), RV/tibia ratio (*C*, *H*), LV/tibia ratio (*D*, *I*), RV/LV + S (*E*, *J*), RVSP (*F*, *K*), in lungs of male (*B–F*) and female (*G–K*) mice treated with vehicle or E3. Elastin staining and percentage of double laminae vessels in lungs of vehicle- or E3-treated mice (*L*, *M*). Scale bars = 100 µm. Male mice are shown as blue bars and females as pink bars, *n* = 10–12 per group. Symbols represent individual data points. Data are represented as mean ± SEM. *P < 0.05; **P < 0.01; ***P < 0.001 (Unpaired *t*-test). RV, right ventricle; LV, left ventricle; S, septum; RVSP, right ventricle systolic pressure.

**Figure 2:**
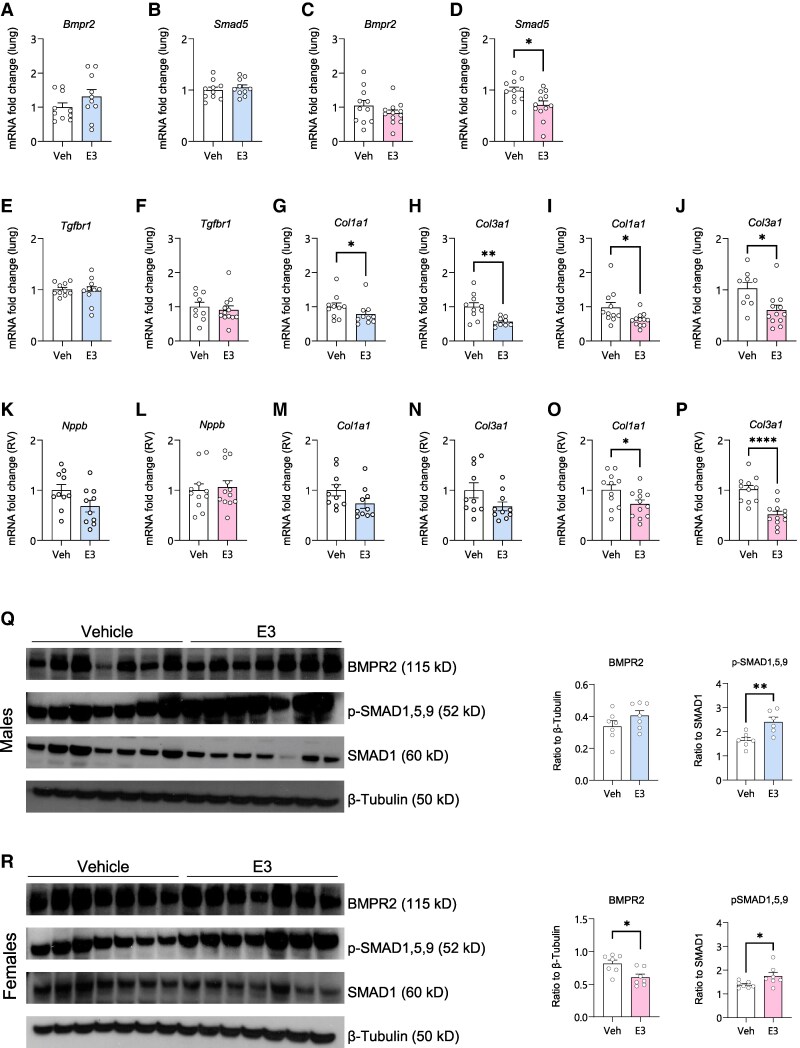
Effect of estriol (E3) on cardiac and pulmonary gene expression. Gene expression analysis of *BMPR2* and *SMAD5* (*A–D*), *TGFBR1* (*E*, *F*), and collagens (*G–J*) in lung tissue. Gene expression analysis of *Nppb* (*K*, *L*) and collagens (*M–P*) in RV tissue. Male mice are shown as blue bars and females as pink bars, *n* = 10–12 per group. Immunoblotting for BMPR2 and phosphoSMAD1,5,9 in the lungs of male (*Q*) and female (*R*) mice treated with E3 or vehicle, *n* = 7 per group. Beta-tubulin was used as the house keeper gene. Symbols represent individual data points. Data are represented as mean ± SEM. *P < 0.05; **P < 0.01; ****P < 0.0001 (Unpaired *t*-test). RV, right ventricle; Bmpr2, bone morphogenetic protein receptor 2; Smad5, small mothers against decapentaplegic 5; TGFBR1, transforming growth factor beta receptor 1; Nppb, Natriuretic Peptide B.

To mimic the age-related decline in estrogen levels observed in human physiology, we used adult mice (20–22 weeks old) and administered them with E3 for two weeks. The physiological effects were assessed by right heart catheterization (*Figure 1A*). In male mice, we did not observe any changes in the final body weight, RVH (RV/LV + S) and RVSP (*Figure 1B–F*). In female mice treated with E3, final body weight and RVSP remained unchanged, whereas RVH was significantly increased, most likely due to the increase in RV weight and decrease in the LV weight (*Figure 1G–K*). Histological analysis revealed no pulmonary vascular remodelling in the lungs of mice treated with E3 (*Figure 1L* and *M*). Real-time PCR analysis of lung tissue revealed no aberrant changes in pulmonary BMPR2 pathway-related genes (*Figure 2A–D*) and pro-fibrotic *TGFBR1* expression (*Figure 2E* and *F*). However, we observed a decrease in the expression of collagens (*Col1a1* and *Col3a1*) in the lungs of both male and female mice treated with E3 (*Figure 2G–J*). mRNA levels of right ventricular B-type natriuretic peptide (*Nppb*), a marker of cardiac stress and remodelling, remained unchanged in both sexes (*Figure 2K* and *L*). While collagen gene expression in the RV was unaltered in male mice (*Figure 2M* and *N*), a significant reduction was observed in females (*Figure 2O* and *P*). Although BMPR2 protein levels were decreased in female mice, BMPR2 signalling was enhanced in the lungs of both male and female mice treated with E3, as demonstrated by increased phosphorylation of SMAD 15,9 protein (*Figure 2Q* and *R*). The sex differences observed in adult mice treated with E3 are summarized in *Table 1*.

**Table 1:** Sex differences in lean and obese mice treated with E3 as compared to the respective vehicle group

Parameters	STD + E3		HFD + E3	
	Males	Females	Males	Females
RV systolic pressure	=	=	=	=
RV hypertrophy	=	↑	=	=
vWAT weight	NA	NA	↓	↓
Pulmonary vascular remodeling	=	=	↓	=
Pulmonary collagen mRNA	↓	↓	=	=
RV collagen mRNA	=	↓	=	=
Visceral adipocyte area	NA	NA	=	↓
Plasma leptin	NA	NA	↓	↓
Plasma triglycerides	NA	NA	=	=

STD, standard diet; HFD, high fat diet; E3, estriol; RV, right ventricle; vWAT, visceral white adipose tissue weight; =, no change; ↑, increase; ↓, decrease; NA, not available.

### E3 decreases the adipose tissue mass and muscularized pulmonary vessels in obese mice

**Figure 3:**
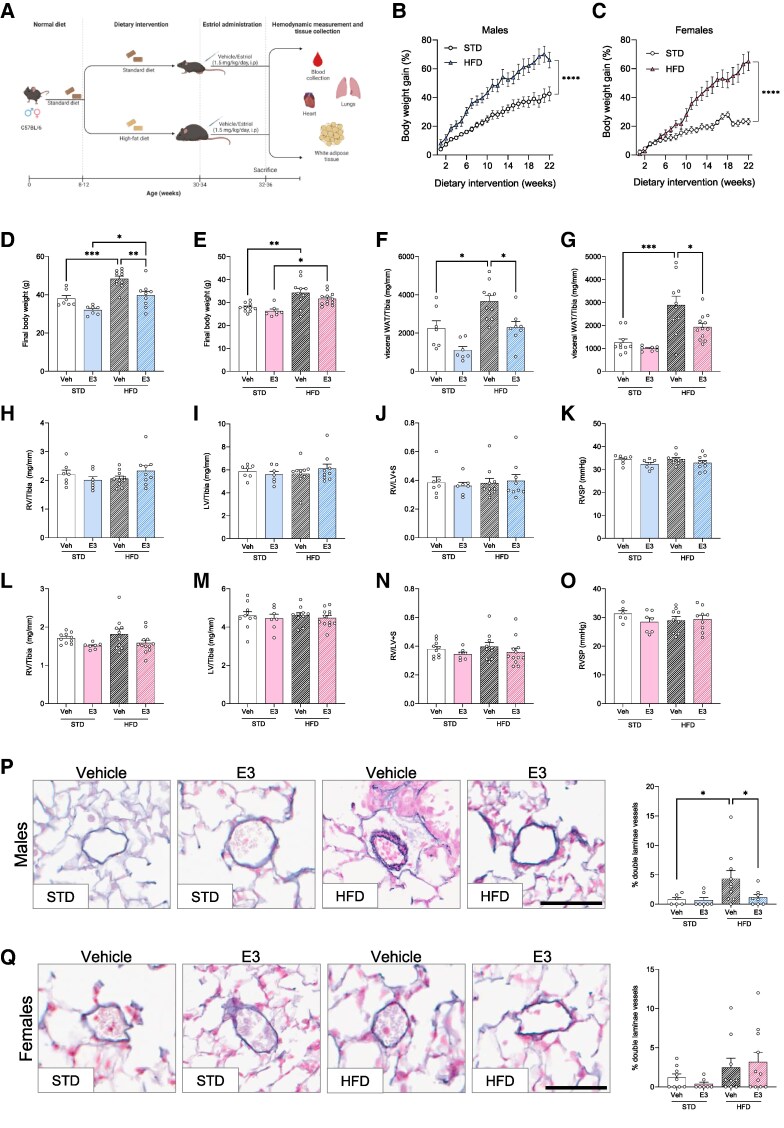
Effect of estriol (E3) on cardiac physiology and hemodynamics in mice on high-fat diet and treated with E3. Schematic representation of the animal experiments (*A*). Percentage of body weight gain in male mice (*B*) and female mice (*C*) on standard (STD) and high-fat diet (HFD) for 22 weeks. Final body weight in males (*D*) and females (*E*). vWAT weight/tibia in males (*F*) and females (*G*). RV/tibia ratio (*H*, *L*), LV/tibia ratio (*I*, *M*), RV/LV + S (*J*, *N*), and RVSP (*K*, *O*) in male (*H–K*) and female (*L–O*) mice. Elastin staining and percentage of double laminae vessels in the lungs of E3- or vehicle-treated mice on STD and HFD (*P*, *Q*). Scale bars = 100 µm. Male mice are shown as blue bars and females as pink bars, *n* = 7–12 per group. Symbols represent individual data points. Data are represented as mean ± SEM. *P < 0.05; **P < 0.01; ****P < 0.0001 (two-way ANOVA followed by Tukey’s correction (*B*, *C*) and one-way ANOVA followed by Šídák correction (*D–Q*). RV, right ventricle; LV, left ventricle; S, septum; RVSP, right ventricle systolic pressure; vWAT, visceral white adipose tissue.

Adipose tissue is an important peripheral site for estrogen production, storage, and metabolism,^22^ especially in aged men and post-menopausal women. To investigate the role of increased adipose tissue in the presence of E3, we fed mice with HFD for 22 weeks before administering E3 (*Figure 3A*). Male and female mice maintained on an HFD exhibited comparable overall body weight gain percentages over a 22-week period, despite females demonstrating a slower rate of weight gain during the initial eight weeks (*Figure 3B* and *C*). No changes were observed in the final body weight of male and female mice on STD treated with E3 (*Figure 3D* and *E*). However, male mice on HFD exhibited a significant reduction in body weight after E3 administration (*Figure 3D*). Moreover, while no changes were observed in lean animals of either sex, there was a reduction in visceral white adipose tissue (vWAT) mass in male and female obese mice treated with E3 (*Figure 3F* and *G*). A combination of HFD and E3 did not affect the ventricular mass, RVH or RVSP in either male (*Figure 3H–K*) or female animals (*Figure 3L–O*). Other cardiac function parameters like contractility index, arterial elastance and cardiac output remained unaltered in obese mice treated with E3 (see Supplementary material online, *Figure S3*). In the male pulmonary vasculature, HFD promoted vascular remodeling which was attenuated by E3 (*Figure 3P*), with no changes observed in female lungs (*Figure 3Q*).

**Figure 4:**
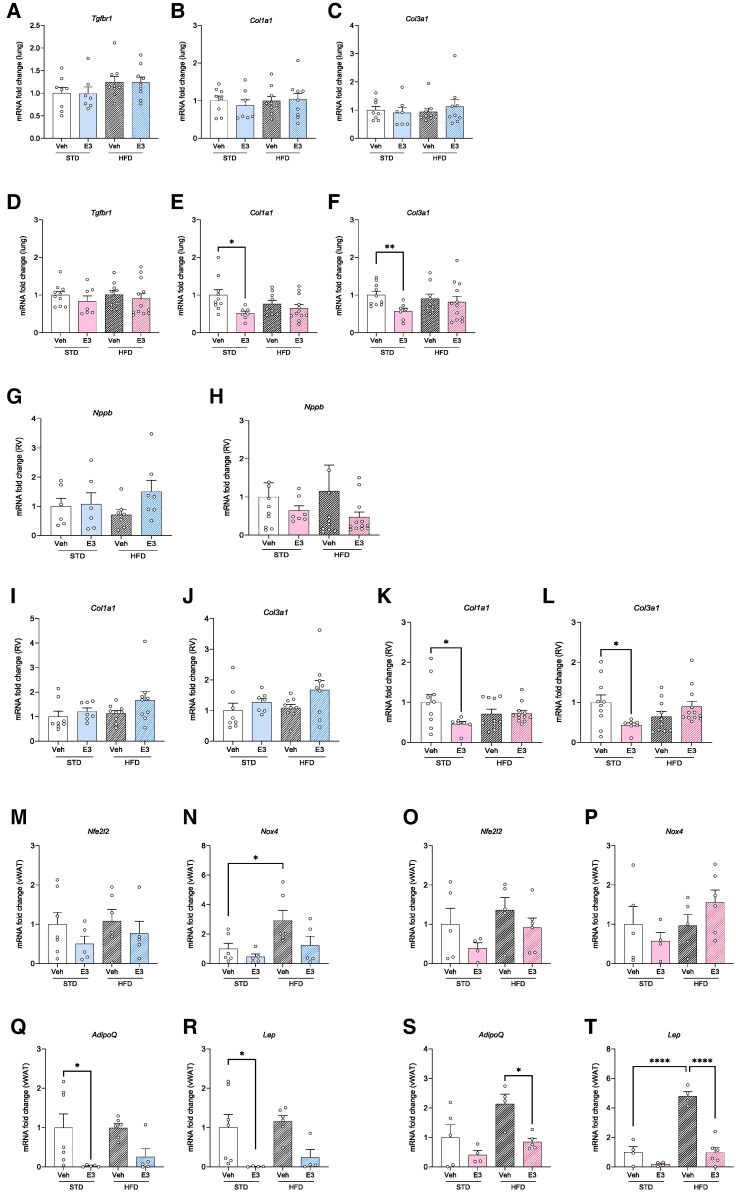
Effect of estriol (E3) on cardiac and pulmonary gene expression in obese mice. Gene expression analysis of pro-fibrotic *Tgfbr1*, *Col1a*1 and *Col3a1* in lung tissue of male (*A–C*) and female (*D–F*) mice. Gene expression analysis of *Nppb* (*G*, *H*) and collagens (*I–L*) in RV tissue of male (*G*, *I*, *J*) and female (*H*, *K*, *L*) mice treated with vehicle or E3, *n* = 7–12 per group. Gene expression analysis of *Nfe2l2* (*M*, *O*), *Nox4* (*N*, *P*), *AdipoQ* (*Q*, *S*), and *Lep* (*R*, *T*) in vWAT tissue of male (*M*, *N*, Q, and *R*) and female mice (*O*, *P*, S, and *T*), *n* = 4–6 per group. Symbols represent individual data points. Data are represented as mean ± SEM. *P < 0.05; **P < 0.01 (one-way ANOVA followed by Šídák correction). Col1a1, collagen type I alpha 1; Col3a1, collagen type III alpha 1; TGFBR1, transforming growth factor beta receptor 1; Nppb, Natriuretic Peptide B; Nfe2l2, Nuclear Factor, Erythroid 2 Like 2; Nox4, NADPH oxidase 4; AdipoQ, adiponectin; Lep, leptin.

To better understand the effect of E3 in the pulmonary vasculature, we analysed the expression of fibrosis-related genes, including estrogen metabolising enzymes, in the lungs and RV of mice on STD and HFD treated with E3. In the lungs of male animals, expression of *Tgfbr1*, *Col1a1*, *Col3a1* (*Figure 4A–C*) and estrogen-related genes (*Cyp1a1*, *Cyp1b1*, *Esr1* and *Esr2,*  Supplementary material online, *Table S1*) remained unchanged. Contrary to this, in the lungs of female mice with STD, E3 decreased the expression of collagens (*Figure 4E* and *F*) while increasing the expression of intracellular ERα (*Esr1)* (see Supplementary material online, *Table S1*). In the RV, *Nppb* mRNA levels were unchanged in both sexes (*Figure 4G* and *H*). Similarly, in lean and obese male mice, RV expression of collagens (*Figure 4I* and *J*) and estrogen pathway genes remained unaltered (see Supplementary material online, *Table S1*). However, in the RV of lean female mice, collagen expression was significantly reduced following E3 treatment (*Figure 4 K* and *L*), accompanied by a notable upregulation of *Cyp1a1*, an estrogen-catabolizing enzyme, and *Esr2* (ERβ) (see Supplementary material online, *Table S1*). These changes were not observed in E3-treated female mice maintained on HFD. Since abnormal reactive oxygen species generation and oxidative stress in adipose tissue have been linked to various abnormalities, we assessed the effect of E3 in modulating this effect in the adipose tissue. In male and female mice treated with E3, the vWAT expression of the *Nfe2l2* gene (encoding nuclear factor erythroid-derived 2-like 2 that regulates antioxidant proteins) did not change (*Figure 4M* and *O*). In male mice, HFD increased the vWAT expression of NADPH oxidase 4 (*Nox4*, regulating beneficial response to reactive oxygen species) and E3 had no significant effect (*Figure 4N*), whereas in female mice *Nox4* expression was unchanged (*Figure 4P*). To assess inflammatory states, we also looked into the adipose tissue expression of adipokine genes, *AdipoQ* and *Lep*. In males with STD, both *AdipoQ* and *Lep* gene expression were significantly decreased after E3 treatment (*Figure 4Q* and *R*), with no changes observed in obese mice. Lean females exhibited no change in *AdipoQ* and *Lep* expression (*Figure 4S* and *T*), in obese mice, E3 significantly reduced the expression of both these genes.

### E3 reduces visceral adipocyte area

**Figure 5:**
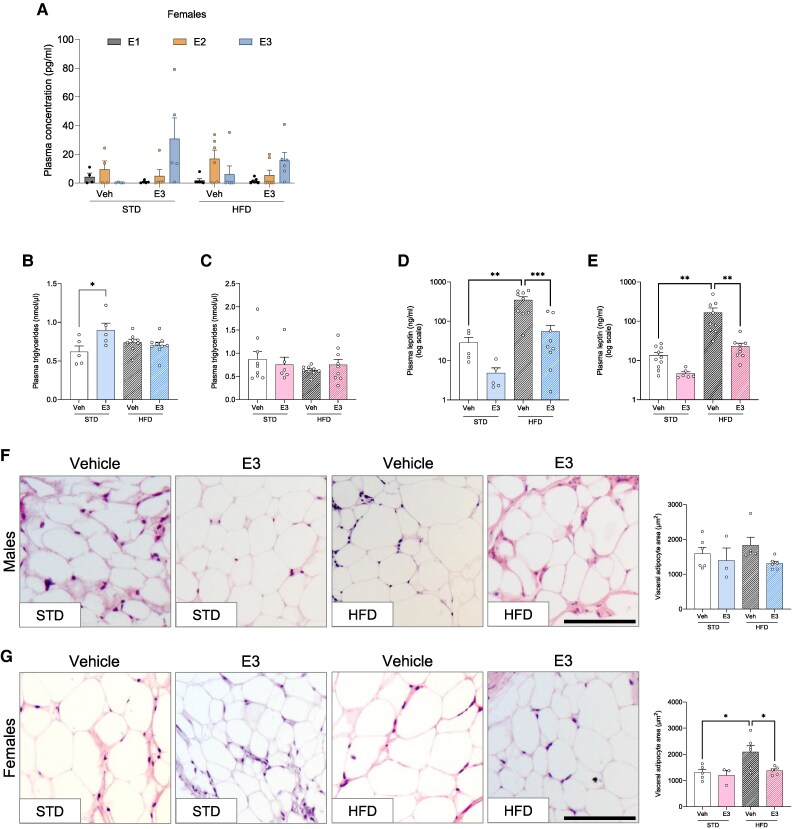
Effect of estriol (E3) on adipocyte inflammation. Plasma levels of E1, E2, and E3 (*A*), triglycerides (*B*, *C*) and leptin (*D*, *E*) in males (*B*, *D*) and females (*C*, *E*). Hematoxylin and eosin staining of visceral adipose tissue and visceral adipocyte area in E3- or vehicle-treated males (*F*) and females (*G*) on standard (STD) or high-fat diet (HFD). Scale bars = 20 µm. *n* = 3–6 per group. Symbols represent individual data points. Data are represented as mean ± SEM. *P < 0.05; **P < 0.01, ***P < 0.001 (one-way ANOVA followed by Šídák correction).

To better understand the effect of exogenous E3 on other estrogens, we measured plasma concentrations of three major estrogens (E1, E2 and E3). Despite its short half-life,^23^ E3 was detected in 60–80% of animals treated with E3 (*Figure 5A* and Supplementary material online, *Figure S4*). In lean females, E3 did not impact the circulating pool of the other two estrogens however, in the female mice on HFD alone, the plasma level of E2 was slightly elevated, which was modestly reduced upon E3 administration (*Figure 5A*). In male mice, the levels of E1 and E2 were below the limit of quantification. To evaluate the peripheral inflammation in obese mice, we measured the circulating levels of triglycerides (TG) and the inflammatory adipokine leptin. Plasma TG levels were increased in lean males with E3, but no elevation was observed in obese males or females in any group (*Figure 5B* and *C*). Circulating levels of pro-inflammatory adipokine leptin were markedly increased in mice maintained on HF, D and E3 treatment significantly decreased these levels in both sexes (*Figure 5D* and *E*). In order to better understand the loss of adipose mass in obese mice treated with E3, we assessed the adipocyte area histologically in vWAT from these animals. E3 administration significantly reduced adipocyte area in females, whereas no change was observed in males following E3 treatment (*Figure 5F* and *G*). The sex differences observed in obese mice treated with E3 are summarized in *Table 1*.

### E3 regulates inflammation and proliferation in human adipocytes and PASMCs

**Figure 6:**
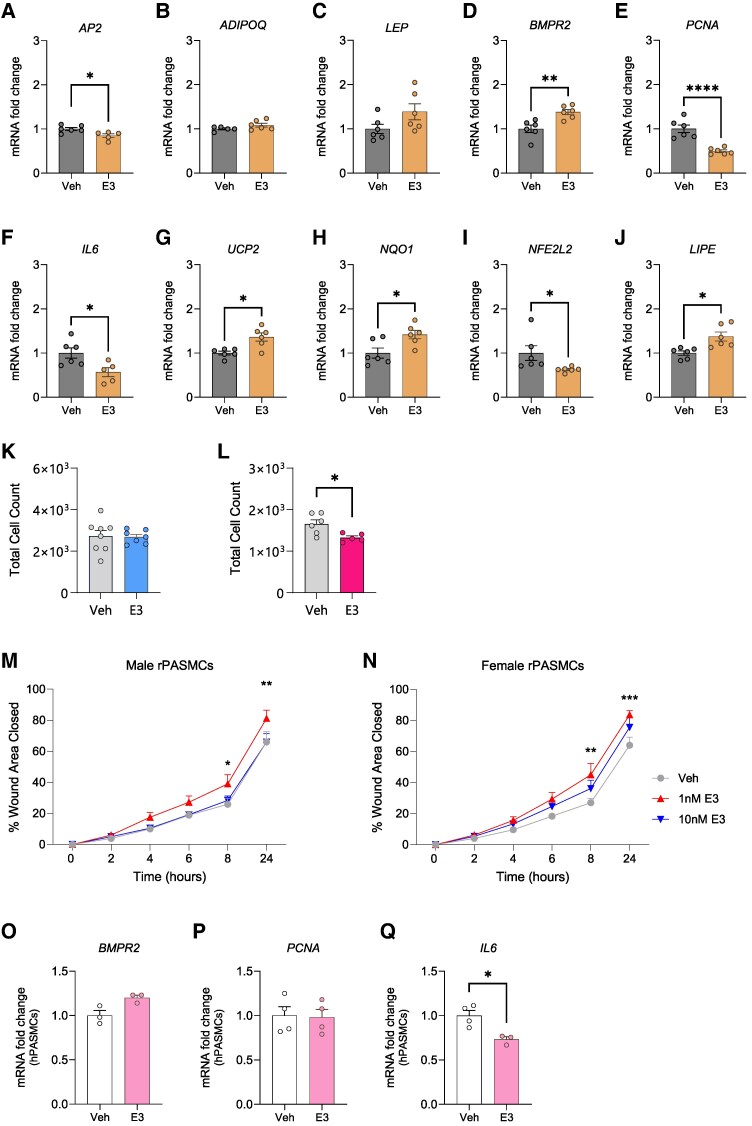
*In-vitro* effect of estriol (E3) on SGBS adipocytes, and PASMCs isolated from rats and humans. Gene expression changes in *AP2* (*A*), *ADIPOQ* (*B*), *LEP* (*C*), *BMPR2* (*D*), *PCNA* (*E*), *IL6* (*F*), *UCP2* (*G*), *NQO1* (*H*), *NFE2L2* (*I*), and *LIPE* (*J*) after stimulation of SGBS adipocytes with 10 nM E3 for 2 h. Total cell count (*K*, *L*) and wound healing assay (*M*, *N*) in pulmonary artery smooth muscle cells isolated from male (*K* and *M*) and female (*L* and *N*) rats. mRNA expression of *BMPR2* (*O*), *PCNA* (*P*), and *IL-6* (*Q*) in PASMCs isolated from female PAH patients. In A-L, symbols represent the mean of technical duplicates for three separate experiments. In M and N, symbols represent mean of cell lines isolated from 3 rats of each sex (**P < 0.01, ***P < 0.001; 1 nm E3 vs. vehicle, 2-way ANOVA with Tukey’s correction). In *O–Q*, symbols represent individual patients. Data are represented as mean ± SEM. *P < 0.05; **P < 0.01, ***P < 0.001 (Unpaired *t*-test in *A–J* and *O–Q*). AP2, adipocyte protein 2; AdipoQ, adiponectin; Lep, leptin; Bmpr2, bone morphogenetic protein receptor 2; PCNA, proliferating cell nuclear antigen; UCP2, uncoupling protein 2; NQO1, NAD(P)H dehydrogenase quinone 1; Nfe2l2, Nuclear Factor, Erythroid 2 Like 2; LIPE, lipase E; r/hPASMCs, rat/human pulmonary artery smooth muscle cells.

To explore the mechanistic effects of E3 on adipocytes, we used non-immortalized human SGBS adipocytes, a well-characterized *in-vitro* model with estrogen receptor expression and robust adipogenic capacity.^24^ SGBS adipocytes treated with E3 for 2 h exhibited decreased mRNA levels of adipocyte protein 2 (*AP2),* while no significant changes were observed in *ADIPOQ* or *LEP* gene expression (*Figure 6A–C*). However, *BMPR2* expression was significantly upregulated (*Figure 6D*), while expression of the inflammatory cytokine *IL6* and the pro-proliferative marker *PCNA* was significantly reduced (*Figure 6E* and *F*). E3 treatment also led to an upregulation of oxidative stress regulators, including uncoupling protein 2 (*UCP2*) and NAD(P)H quinone dehydrogenase 1 (*NQO1,* *Figure 6G* and *H*), alongside a reduction in *NFE2L2* expression (*Figure 6I*). In addition, E3 induced a significant increase in the expression of the triglyceride-hydrolysing enzyme lipase E (*LIPE*, *Figure 6J*).

Consistent with the anti-inflammatory effects of E3 in adipocytes, cell proliferation was significantly reduced in female rat PASMCs treated with E3 (*Figure 6K* and *L*). Moreover, wound healing was moderately enhanced in PASMCs from both male and female rats (*Figure 6M* and *N*). In PASMCs from female PAH patients (see Supplementary material online, *Table S2*), E3 treatment led to a moderate, but non-significant increase in *BMPR2* gene expression, accompanied by a reduction in *IL6* mRNA levels, while *PCNA* expression remained unchanged (*Figure 6O* and *P*).

### ERα mediates E3-induced signalling in rat PASMCs

To elucidate the mechanism through which E3 exerts its functions, rat PASMCs were stimulated with E3 in the presence of selective estrogen receptor antagonists: Methyl-Piperidinopyrazole (MPP, ERα antagonist), 4-[2-Phenyl-5,7-*bis*(trifluoromethyl)pyrazolo[1,5-*a*]pyrimidin-3-yl]phenol (PHTPP, ERβ antagonist), and G15 (GPER antagonist). The expression levels of *BMPR2*, *SMAD1*, and *SMAD4* were significantly reduced following stimulation with E3 alone or in combination with PHTPP and G15. However, this reduction was not observed when E3 was administered in conjunction with the ERα antagonist MPP, suggesting that E3 exerts its effects through ERα receptors (see Supplementary material online, *Figure S5*).

## Discussion

E3, a natural estrogen produced during pregnancy with weaker estrogenic activity and different bioavailability than E2, has an unclear role in the adult cardiopulmonary-vascular system. Here, we demonstrate for the first time that E3 reduces vWAT mass by attenuating adipocyte inflammation and proliferation. Furthermore, our findings suggest that E3 may confer protective effects on the pulmonary vascular system by inhibiting cell proliferation and promoting wound healing.

Obesity has been recognized as a pathological condition that contributes to the development of CVD by (1) promoting a chronic inflammatory and prothrombotic state, and (2) exacerbating established cardiovascular risk factors such as diabetes, hypertension, and dyslipidemia. Adipose tissue, beyond serving as an energy reservoir, functions as a metabolically active endocrine organ and is a known site of estrogen synthesis and storage. Estrogens have been implicated in several CVDs such as PAH, heart failure and thromboembolic disorders.^25^ Our previous studies showed elevated estrogen and metabolites in PAH patients, with 16OHE1 promoting pulmonary hypertension^26^ and also contributing to vascular remodelling in obese mice.^13^ Building upon these findings, the current study aimed to investigate how obesity, in conjunction with another estrogen metabolite E3, influences the cardiovascular and pulmonary-vascular systems.

In the initial experiment to mimic human physiology of declining estrogens with age, we treated adult mice with E3 for a duration of two weeks to evaluate its physiological effects. Mice of both sexes treated with E3 did not have unfavourable changes in physiological/hemodynamic parameters or vascular alterations in the lung. Female mice treated with E3 exhibited increased RVH, potentially indicative of RV abnormality, however this increase in RVH could be attributable to the significant reduction in the LV weight. Fibrosis is one of the hallmarks of RV failure and animal studies suggest that ventricular fibrosis contributes to the switch from adaptive to maladaptive remodeling.^27^ Estrogens have been shown to counteract the TGFβ-dependent activation of fibroblasts^28^ and a direct interaction between downstream SMAD2/3 and ESR1 has been described.^29^ In our study, E3 administration significantly decreased collagen gene expression both in the lungs and the RV of male as well as female lean mice. Additionally, an increase in BMPR2 signalling was detected at the protein level in the lungs of these mice, emphasising the role of E3 in modulating pro-fibrotic gene expression. In the second experimental setup, where mice were maintained on HFD followed by E3 administration, this decrease in collagen expression was limited to lean animals. It has been described that obesity increases the formation of nonreducible collagen cross-links in mice^30^ which, in part, might explain no effect of E3 on collagen expression in obese mice. Male mice on HFD also exhibited moderately increased vascular remodeling which was attenuated after E3 treatment.

Visceral adipose tissue has been strongly associated with metabolic dysfunction due to adipocyte hypertrophy and elevated proinflammatory cytokines.^31^ Exogenous estrogens show protective metabolic effects by modulating adipose tissue distribution.^32^ Consistent with this, we observed reduced vWAT mass in obese animals treated with E3, mirroring estrogen’s role in body weight regulation in humans.^33^ Mechanistically, E3 treatment decreased visceral adipocyte area in E3-treated mice, suggesting an additional pathway through which estrogen metabolites may influence body weight.

While estrogens have been implicated in disease aetiology, they also play an immune-supportive role. E2 was shown to inhibit NF-κB activation in mouse monocytes,^34^ rat SMCs,^35^ as well as human aortic endothelial cells,^36^ while its metabolites, such as 2-hydroxyestrogens, mediate anti-inflammatory responses.^37,38^ E3 has also demonstrated beneficial effects in autoimmune diseases, including experimental autoimmune encephalomyelitis,^34^ collagen-induced arthritis,^39^ and multiple sclerosis,^40^ by modulating cytokines and favourably altering the repertoire of circulating immune cells. In SGBS adipocytes, E3 reduced pro-proliferative (*PCNA*) and pro-inflammatory (*IL6*) gene expression while upregulating antioxidative genes (*NQO1*), underscoring its anti-inflammatory effects. Contrary to our findings in adipocytes, E3 increased *IL6* expression in murine macrophages during endotoxemia^41^ suggesting that E3 may exert differing effects depending on the cell type.

This study is the first to quantify plasma E3 levels in mice using an LC-MS/MS-based assay. Despite the high metabolic clearance rate, we were able to detect E3 in lean and obese mice of both sexes one day after the final injection. A plausible explanation for this observation could be the presence of a sufficient circulating pool of estrogens, potentially reducing the necessity for estrogen-metabolising enzymes to metabolize E3. In lean mice, exogenous E3 administration did not alter peripheral E1 and E2 levels. However, in obese mice treated with E3, circulating E2 levels were modestly reduced compared to the vehicle-treated group. Plasma concentration of E3 in obese mice treated with E3 was slightly less compared to lean E3-treated mice. This reduction could be due to the differences in adipose-specific metabolism in lean and obese animals.^42^ Notably, obesity itself appeared to slightly elevate the circulating pool of E2 in female mice without significantly affecting endogenous E3 levels. This elevation in E2 may be attributable to increased aromatase activity in visceral adipose tissue, which is known to convert androgens to estrogens under adipose conditions.^43^

Leptin, a pro-inflammatory adipokine released from white adipose tissue,^44^ is shown to be an important predictor of metabolic dysfunction in obese patients.^45^ Irrespective of sex, plasma leptin levels were elevated in HFD-fed mice but were significantly reduced following E3 treatment. This reduction in leptin could be attributed to vWAT loss, as leptin release is directly proportional to the amount of stored fat.^46^ Notably, despite increased vWAT, HFD-fed mice showed no rise in plasma TG levels. E3 may have promoted TG storage in adipose tissue, supported by *in-vitro* findings showing E3-induced upregulation of *LIPE*, which hydrolyses stored TGs into free fatty acids.^47^

Excessive proliferation is a hallmark of pulmonary vascular remodelling in PAH. In experimental models, E2 shows varied effects: it protects against monocrotaline-induced PAH in female rats^48^ but fails to prevent PAH in a severe angio-proliferative model, where females develop more severe disease than males.^49^ Additionally, we found that estrogen inhibition (via anastrozole^50^ or ovariectomy^3,51^) reverses experimental PH in female rodents. However, the role of estrogen metabolite E3 in the pulmonary vasculature remains unexplored. To this end, we performed proliferation and wound healing assays in rat PASMCs. E3 significantly decreased PASMCs' proliferation and enhanced wound healing, reflecting the interplay of inflammation, proliferation, and remodelling during normal wound healing. Reduced SMC proliferation may underlie the diminished vascular remodelling seen in E3-treated mice. Beyond having cellular effects, E3 may influence other immune molecules, including chemokines and adhesion molecules.

Both lean and obese mice treated with E3 exhibited a reduction in vWAT weight, smaller adipocyte area, and lower plasma leptin concentration, indicating reduced adipocyte inflammation. In a Japanese study, E3 was effective in decreasing total cholesterol and TGs as well as increased high density lipoprotein concentrations in elderly women.^52^ With regards to other cardiopulmonary complications, Toy et al report that an ester of E3 (estriol succinate) may have less thrombotic potential than synthetic estrogens.^53^ So far, most of the studies involving E3 have been focused on neurodegenerative/autoimmune disorders or menopause-related conditions. To determine whether E3 exerts protective cardiac effects, comprehensive studies are needed, including validation in experimental models and assessment of circulating E3 levels in patients.

We identified some noteworthy sex differences in our study, as summarized in *Table 1*. Notably, pulmonary vascular remodelling was reduced in E3-treated obese males, whereas no significant changes were observed in females. This lack of effect in females could be attributed to the presence of higher circulating estrogens and pre-existing ERα activation, which could diminish the additional impact of exogenous E3. Additionally, E3 administration resulted in a reduction in visceral adipocyte area in females, while no difference was seen in males. Given that selective activation of ERα in adipose tissue has been shown to induce lipolysis,^54^ the lower ERα expression in males^55^ may contribute to a reduced sensitivity to E3-induced lipolysis.

## Conclusions

This is the first report demonstrating that E3 modulates obesity by influencing adipocyte inflammation. We report that E3 administration decreases fat depot mass in obese mice primarily by decreasing the adipocyte size. *In-vitro*, E3 stimulation attenuates inflammatory and proliferative properties in human adipocytes and PASMCs. Furthermore, our findings suggest that E3 exerts non-detrimental, potentially anti-inflammatory effects on the pulmonary vasculature.

Despite several strengths, this study has limitations. The urinary concentrations of estrogen metabolites were not measured, which would have provided a more comprehensive understanding of estrogen metabolism in animals on HFD. However, to our knowledge, the assay for measuring E3 in mouse urine has yet to be developed. Assessing cholesterol and insulin levels would provide additional insight into the metabolic profile of obese mice, but limited blood volume necessitated prioritizing measurement of triglycerides, leptin, and estrogens to address our primary objectives. Although female mice were group-housed to promote cycle synchronisation,^50^ residual variability in cycle stage cannot be fully excluded. The endogenous estrogen milieu may synergize with exogenous E3, potentially enhancing anti-inflammatory responses. Furthermore, the expression and activity of ERs fluctuate across the oestrous cycle,^56^ potentially modulating E3-induced effects. Therefore, considering the oestrous cycle is essential when evaluating the efficacy and mechanisms of estrogen-based therapies. Ours is among the first studies to investigate the physiological effects of E3, and therefore, the optimal duration of *in-vivo* treatment was not predefined. We relied on previous studies showing that estrogenic compounds elicit measurable molecular and physiological responses within comparable time frames^57,58^. Nevertheless, longer exposure periods may better reflect hormonal changes in humans. Finally, our study was exploratory, and the causality and translational relevance need to be confirmed in further studies.

## Lead author biography


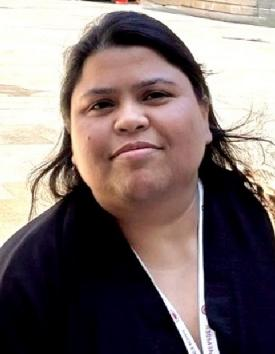


Dr Smriti Sharma is a translational scientist focused on the immunological mechanisms underlying cardiovascular diseases, with particular emphasis on pulmonary hypertension and thrombosis. She completed her PhD in Vascular Biology from the Medical University of Vienna, Austria, followed by a postdoctoral stay at the University of Strathclyde in Glasgow, UK, where she expanded her focus to understanding sex differences in cardiopulmonary disorders.

## Supplementary Material

oeag001_Supplementary_Data

## Data Availability

All data relevant to this article are in the article or in its supplemental material.
