# Correction to: Estriol attenuates visceral adiposity and pulmonary artery smooth muscle cell proliferation via ERα-mediated signalling

**DOI:** 10.1093/ehjopen/oeag122

**Published:** 2026-08-10

This is a correction to: Smriti Sharma, Joshua P Dignam, Gregor Aitchison, Rosemary Gaw, Ioannis Stasinopoulos, Ayman Gebril, Martin Wabitsch, Ruth Andrew, Margaret R MacLean, Estriol attenuates visceral adiposity and pulmonary artery smooth muscle cell proliferation via ERα-mediated signalling, *European Heart Journal Open*, Volume 6, Issue 1, January 2026, oeag001, https://doi.org/10.1093/ehjopen/oeag001

In the originally published version of this manuscript, author Ioannis Stasinopoulos’ name was misspelled.

This error has been corrected.

