# Supplementary material for: Estriol attenuates visceral adiposity and pulmonary artery smooth muscle cell proliferation via ERα-mediated signalling
Source: Eur Heart J Open. 2026 Jan 20;6(1):oeag001. doi: 10.1093/ehjopen/oeag001 (PMC12870853; doi:10.1093/ehjopen/oeag001)
Supplement: oeag001_Supplementary_Data [file oeag001_supplementary_data.docx]

**SUPPLEMENTAL DATA**

**Estriol attenuates visceral adiposity and pulmonary artery smooth muscle cell proliferation via ERα-mediated signaling**

**Short title:** Estriol, obesity and pulmonary vascular remodeling

Smriti Sharma^1,2^, Joshua P Dignam^1,3^, Gregor Aitchison^1^, Rosemary Gaw^1^, Ioannis Stasinopolous^4^, Ayman Gebril^1^, Martin Wabitsch^5,6^, Ruth Andrew^4^, Margaret R MacLean^1^

^1^Strathclyde Institute of Pharmacy and Biomedical Sciences, University of Strathclyde, Glasgow, Scotland, UK. ^2^Department of Internal Medicine II, Cardiology, Medical University of Vienna, Vienna, Austria. ^3^Centre for Microvascular Research, William Harvey Research Institute, Barts and The London School of Medicine and Dentistry, Queen Mary University of London, Charterhouse Square, London, UK. ^4^Center for Cardiovascular Science, Queen's Medical Research Institute, University of Edinburgh, Edinburgh, Scotland, UK. ^5^Division of Pediatric Endocrinology and Diabetes, Department of Pediatrics and Adolescent Medicine, Ulm University Medical Center, Ulm, Germany. ^6^German Center for Child and Adolescent Health (DZKJ), partner site Ulm, Ulm, Germany.

**Correspondence to:**

Smriti Sharma, PhD. Department of Internal Medicine II, Division of Cardiology. Medical University of Vienna, Waehringer Guertel 18-20. A-1090 Vienna, Austria. Tel: +43 1 40400 73520, Fax: +43 1 40400 42160. Email: smriti.sharma@meduniwien.ac.at

**Supplemental Methods**

**Lung Histopathology**

A key pathological feature of PH is vascular remodeling involving proliferation and phenotypic transformation of endothelial cells and smooth muscle cells. In order to assess vascular remodeling in mice, lung sagittal sections (5µm) were stained with Miller’s elastin stain and counterstained with 1% neutral red ^1^. Small pulmonary arteries with an external diameter <80 µm were microscopically analyzed in a blinded fashion to assess vascular remodeling (90-100 vessels per mouse). Small arteries close to the bronchus or large muscularized vessels were excluded. Remodeled arteries were identified by the presence of double-elastic laminae and are represented as percentage of total vessels counted.

**Triglyceride measurement**

Mouse plasma triglyceride level was measured using a commercial kit (Abcam, UK). Briefly plasma samples were diluted as appropriate, and triglyceride levels were measured in a colorimetric assay as per manufacturer’s protocol.

**Immunoblotting**

Lung and RV tissues were homogenized in 300-400 μL of T-PERTM Tissue Protein Extraction Reagent (ThermoFisher Scientific, UK) containing Halt Protease Inhibitor Cocktail. Tissue lysate was centrifuged at 10,000 rpm for 15 min at 4 °C to pellet the debris and supernatant was transferred to fresh tubes. Protein concentration was determined using Bicinchonic acid assay (BCA assay; Pierce, ThermoFisher, UK). 25 µg of protein was separated on NuPAGE Bis-Tris 4-12% Polyacrylamide Gels (Invitrogen, UK) and transferred onto a 0.45 μm polyvinylidene difluoride (PVDF) membrane (Pierce®, ThermoFisher Scientific, UK). Membranes were blocked in Superblock T20 (TBS) Blocking Buffer (ThermoFisher Scientific, UK) for 1 hour at room temperature before incubating overnight with the primary antibodies: BMPR2, pSMAD1/5/9, SMAD1, and β-tubulin. Densitometric analysis was performed with CLIQS 1D, Version 1.5.170 (TotalLab, UK). The level of target proteins (BMPR2, pSMAD1/5/9, and SMAD1) was normalized to the expression of β-tubulin, probed on the same blot. In the next step, phosphorylation level of SMAD1,5,9 protein was further normalized to the levels of total SMAD1 protein. BMPR2 protein level is represented as “ratio to β-tubulin” whereas phospho-SMAD1,5,9 level is represented as “ratio to SMAD1”.

**Quantitative real time PCR**

Total RNA was extracted from RV and lung tissues using Reliaprep RNA Miniprep system according to manufacturer’s instructions. RNA yield was quantified using a NanoPhotometer N60, (Implen GmbH) and cDNA was synthesized using TaqMan Reverse Transcription Reagents (Applied Biosystems). Quantitative real time PCR was performed on ViiA 7 Real-Time PCR System (Applied Biosystems, UK). GAPDH and ACTB were used as housekeeper genes for RV and lung tissues respectively. TBP and RPL19 was used as housekeeper genes for vWAT. For experiments with rat PASMCs, ACTB was used as a housekeeper. The fold change for every gene was obtained using the 2-ΔΔCt method and expressed relative to vehicle control as appropriate. Details of TaqMan assays used for gene expression studies are provided in Supplemental Table S3.

**Human Simpson-Golabi-Behmel syndrome cells**

Human Simpson-Golabi-Behmel syndrome (SGBS) preadipocyte cells were kindly provided by Prof. Martin Wabitsch (Ulm University Medical Center, Germany). Pre-adipocytes were cultured in DMEM/F12 media with 10% FBS until 90% confluent. Differentiation into adipocytes was initiated started by washing cells with PBS and changing to a serum- albumin- free differentiation media (day 0) as described previously ^2^. Within a few days cells start to accumulate lipids and after 2 weeks, >90% of the cells are fully differentiated exhibiting significant triglyceride accumulation. Adipocyte differentiation was also visually confirmed by Oil Red O staining as described below and shown in **Supplemental Figure S1**. On day 14 of differentiation, cells were treated with 10 nM E3 for 2, 8, and 24 hours to determine the optimal time point for detecting gene expression changes in inflammatory markers and markers of adipocyte differentiation and function (**Supplemental Figure S2**). Based on these experiments, 2-hour time point was selected for subsequent experiments.

**Oil Red O staining**

Adipocytes were examined visually with oil red O staining at different days of differentiation (day 0, day 7, and day 14) (**Supplemental Figure S1**). Cells were fixed for 30 min in 10% neutral buffered formalin (Sigma-Aldrich, UK) before washing 3 times with PBS. 60% isopropanol was added to the fixed cells and incubated for 5-8 mins followed by addition of Oil Red O working solution for 20 min. Excess stain was washed and cells were counterstained with Hematoxylin before washing thoroughly with water. Cells were visualized and images acquired on a EVOSTM XL Core Imaging System (Thermo Fisher, UK).

**Human pulmonary artery smooth muscle cells**

Experimental procedures using human cells conform to the principles outlined in the Declaration of Helsinki. Human pulmonary artery smooth muscle cells (PASMCs) were harvested from PAH patients at autopsy and provided by Professor Nick Morrell (University of Cambridge, UK) with ethical permission (**Supplemental Table S2**). Primary cultures were isolated from the small distal pulmonary arteries (<1mm external diameter). For the proliferation assay, cells from female PAH patient were seeded in 6-well plates at 2 x 10^5^ cells/ well.

For all cell culture experiments with E3 stimulation, phenol-red free media with charcoal-stripped FBS was used to avoid any confounding estrogenic activity.

**Rat PASMCs isolation and characterization**

Male and female Sprague-Dawley rats (Envigo, UK) aged 11-13 weeks weighing 320-344g (males) and 210-265g (females) were euthanised by CO2 inhalation (BOC, UK) in accordance with Schedule 1 of the Animals (Scientific Procedures) Act 1986. The heart and lungs were excised, rinsed in sterile PBS, and placed in 2 mL sterile Ham’s F-12 nutrient mixture media (Gibco, UK). The pulmonary arteries were incubated overnight in a culture dish containing digestive mix (10 mg bovine serum albumin (BSA; Sigma-Aldrich, UK), 5 mg collagenase type I (Sigma-Aldrich, UK), 0.6 mg elastase type III (Sigma-Aldrich, UK), 1.8 mg soybean trypsin inhibitor (Sigma-Aldrich, UK) in 10 mL F-12 Ham’s media. Vessels were chopped into small pieces, then re-suspended in the media followed by filtering the cell solution through a 100 μM nylon cell strainer (FALCON, New York, U.S). Cell pellet was re-suspended in 5 mL DMEM containing 20% FBS and 1% antibiotic antimycotic solution. T-25 flasks were coated with 1 mL gelatin (bovine; Sigma-Aldrich, UK), prior to plating the cells.

For immunocytochemistry characterization, cells were seeded in 12-well plates onto collagen coated coverslips at a density of 1 x 10^5^ cells/well. The cells were left to adhere for 24 hours then washed twice with PBS. Cells were fixed in 10% neutral buffered formalin (Sigma-Aldrich, UK) and permeabilized in PBS-Tween-Triton X-100 followed by blocking in 4% BSA, 10% goat serum (Sigma-Aldrich, UK). Primary antibodies: anti-alpha smooth muscle actin (ab5694; Abcam, UK) and anti-vimentin (ab8978; Abcam, UK) were prepared in antibody dilution buffer (4% BSA in PBS-TT) and incubated with cells overnight at 4 °C. The following secondary antibodies were prepared: goat anti-rabbit IgG Alexa Fluor 488 (A-11008; Invitrogen, UK) and goat anti-mouse Alexa Fluor 594 (A11005; Invitrogen, UK) and incubated with cells in the dark for 1 hour at room temperature. Cover slips were mounted on slides with the Vectashield Antifade Mounting Medium containing DAPI as a nuclear stain; Novus Biologicals, UK). The cells were imaged using a Nikon Eclipse E600 fluorescence microscope (Nikon, Japan), images were recorded using WinFluor Software (V3.8.5.; University of Strathclyde, UK) and analyzed using ImageJ (v1.53e; National Institutes of Health, Bethesda, US).

**Stimulation with estrogen receptor antagonists**

Male rat PASMCs were seeded at 3 x 10^5^ cells/well in a 6-well plate and grown till 90% confluent. Quiescent media was added for next 24 hours before returning cells to charcoal-stripped 1% FBS and stimulated for 24 hours with E3 alone or in the presence of 1 μM concentration of the following estrogen receptor antagonists: the ERα antagonist MPP dihydrochloride (Tocris, UK), the ERβ antagonist PHTPP (Tocris, UK) and the GPER antagonist G-15 (Tocris, UK). Following stimulation, cells were collected in QIAzol Lysis Reagent (Qiagen, UK) for total RNA isolation.

**Proliferation assay**

Cells were seeded in a 6-well plate at a density of 2 x 10^5^ cells per well and incubated until ~ 50% confluent before adding the quiescent media (phenol-red free DMEM with 0.2% charcoal-stripped FBS) for 24 hours. After 24 hours cells were returned to charcoal-stripped 1% FBS and stimulated with 10 nM E3 for 48 hours. 10 nM ethanol served as a vehicle control and phenol red-free DMEM containing 10% charcoal stripped FBS was used as a positive control. Cells were trypsinized, resuspended in PBS and kept on ice until assayed. Trypan blue was added right before counting and every well was assayed in duplicate (both sides of the Countess cell counting slide; Life Technologies, UK). The average total cell count from both chambers of the slide was calculated for each sample and plotted using GraphPad Prism 10.1.1.

**Wound migration assay**

Male and female rat PASMCs were seeded in gelatin-coated 6-well plates (3 x10^5^ cells/well) and cultured until ~90-95% confluent. The cells were quiesced in phenol-red free DMEM with 0.2 % charcoal-stripped FBS for 24 hours before stimulating with 1 nM or 10 nM E3. Four images were taken per well at 0, 2, 4, 6, 8, and 24 hours after stimulation using an EVOSTM XL Core Imaging System (ThermoFisher, UK) with EVOS® LPlan PH2 4x/0.13 objective (Life Technologies, UK). Wound area and background area in each image was measured using ImageJ. The average wound area for each well at every time point was calculated and made relative to the background image area to normalize for differences in wound thickness. The average wound area relative to the background area at 0 hours was set as 100%, and the relative percentage of wound area remaining was calculated. As the percentage wound area remaining is inversely proportional to cell migration, the percentage wound area closed was calculated by subtracting the area remaining from 100%.

**Liquid Chromatography with tandem Mass Spectrometry analysis**

Mouse plasma samples were subjected to Liquid Chromatography with tandem mass spectrometry (LC-MS/MS) analysis using the method of Denver et al^3^ with slight modifications. Briefly, automated extraction was performed using a Biotage Extrahera liquid handling robot. Samples and calibration standards were pre-treated by dilution with formic acid (1% v/v, 0.3 mL). Steroids were eluted in 100% methanol and collected in 96-well plates with 700 μL glass inserts Waters (Milford, MA, US). Extracts were reduced to dryness under oxygen-free nitrogen (40 °C) and residues derivatized as previously described except using 0.1 M sodium bicarbonate. 25 µL sample was injected and analysis was performed as previously reported ^3^ on a QTrap 6500 mass spectrometer (Sciex, Warrington, UK) but coupled to an Acquity I class UHPLC (Waters, Manchester, UK).

**Supplemental Tables and Figures**

**Supplemental Tables**

**Supplemental Table S1.** Gene expression analysis of lung and right ventricle tissue from lean and obese mice treated with E3 as compared to the respective vehicle group

| **Pathway** | **Gene** | **STD+E3** | | **HFD+E3** | |
| --- | --- | --- | --- | --- | --- |
|  |  | male | female | male | female |
| **Lung** |  |  |  |  |  |
| BMPR2 signaling | *Bmpr2* | ns | ns | ns | ns |
|  | *Smad5* | ns | ns | ns | ns |
| Fibrosis-related | *TGFBR1* | ns | ns | ns | ns |
|  | *Col1a1* | ns | **↓** | ns | ns |
|  | *Col3a1* | ns | **↓** | ns | ns |
| Estrogen metabolism | *Cyp1a1* | ns | ns | ns | ns |
|  | *Cyp1b1* | ns | ns | ns | ns |
|  | *Esr1* | ns | **↑** | ns | ns |
|  | *Esr2* | ns | ns | ns | ns |
| **Right Ventricle** |  |  |  |  |  |
| Cardiac stress | *Nppb* | ns | ns | ns | ns |
| BMPR2 signaling | *Bmpr2* | ns | ns | ns | ns |
| Fibrosis-related | *Col1a1* | ns | **↓** | ns | ns |
|  | *Col3a1* | ns | **↓** | ns | ns |
| Estrogen metabolism | *Cyp1a1* | ns | **↑** | ns | ns |
|  | *Cyp1b1* | ns | ns | ns | ns |
|  | *Esr1* | ns | **↑** | ns | ns |
|  | *Esr2* | ns | **↑** | ns | ns |
| Adipocyte inflammation | *AdipoQ* | **↓** | ns | ns | **↓** |
|  | *Lep* | **↓** | ns | ns | **↓** |

STD; standard diet, HFD; high fat diet, E3; estriol, Bmpr2; bone morphogenetic protein receptor 2, Smad5; Mothers Against Decapentaplegic Homolog 5, Col1a1; collagen type 1 alpha 1 chain, Col3a1; collagen type 3 alpha 1 chain, Cyp1a1; Cytochrome P450 Family 1 Subfamily A Member 1, Cyp1b1; Cytochrome P450 Family 1 Subfamily B Member 1, Esr1; estrogen receptor 1, Esr2; estrogen receptor 2, TGFBR1; transforming growth factor receptor 1, Nppb; B-type natriuretic peptide, AdipoQ; adiponectin, Lep; leptin, ns; not significant.

**Supplemental Table S2.** Demographics of PAH donors for pulmonary artery smooth muscle cells

| **Study ID** | **Sex** | **Age** | **Indication** |
| --- | --- | --- | --- |
| PAH 1 | F | 45 | APAH (congenital heart disease) |
| PAH 2 | F | 52 | Secondary PAH associated with septal defect |
| PAH 3 | F | 30 | HPAH (*BMPR2* mutation) |
| PAH 4 | F | NA | HPAH (*BMPR2* mutation) |

PAH; pulmonary arterial hypertension, APAH; associated pulmonary arterial hypertension, F; female, BMPR2; bone morphogenetic protein receptor 2, NA; not available.

**Supplemental Table S3.** Details of TaqMan assays used for gene expression studies.

| Gene | Species | Assay ID |
| --- | --- | --- |
| *Actb* | Human | Hs01060665_g1 |
| *Tbp* | Human | Hs00427620_m1 |
| *AdipoQ* | Human | Hs00977214_m1 |
| *Nox4* | Human | Hs01379108_m1 |
| *Nfe2l2* | Human | Hs00975961_g1 |
| *Lep* | Human | Hs00174877_m1 |
| *PCNA* | Human | Hs00427214_g1 |
| *IL6* | Human | Hs00174131_m1 |
| *UCP2* | Human | Hs01075227_m1 |
| *Bmpr2* | Human | Hs00176148_m1 |
| *LIPE* | Human | Hs00943410_m1 |
| *cyp1a1* | Human | Hs01054797_g1 |
| *cyp19a1* | Human | Hs00903413_m1 |
| *Gapdh* | Mouse | Mm99999915_g1 |
| *Actb* | Mouse | Mm00667939_s1 |
| *Bmpr2* | Mouse | Mm00432134_m1 |
| *Smad3* | Mouse | Mm01170760_m1 |
| *Col1a1* | Mouse | Mm00801666_g1 |
| *Col3a1* | Mouse | Mm00802300_m1 |
| *AdipoQ* | Mouse | Mm04933656_m1 |
| *Nox4* | Mouse | Mm00627696_m1 |
| *Nfe2l2* | Mouse | Mm00477784_m1 |
| *Lep* | Mouse | Mm00434759_m1 |
| *RPL19* | Mouse | Mm02601633_g1 |
| *Actb* | Rat | Rn00667869_m1 |
| *Bmpr2* | Rat | Rn01437214_m1 |
| *Smad1* | Rat | Rn00565555_m1 |
| *Smad4* | Rat | Rn00570593_m1 |

**Supplemental Figures**

**
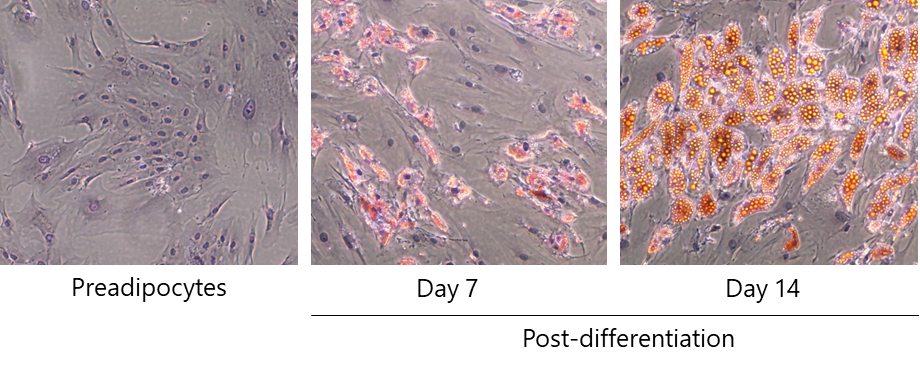
**

**Supplemental Figure S1. Oil red O staining in undifferentiated and differentiated SGBS cells.** Oil red O staining in undifferentiated preadipocytes and mature adipocytes day 7 and day 14 post differentiation.

**Supplemental Figure S2. Effect of E3 on SGBS differentiation and function.** Gene expression analysis of markers of adipocyte differentiation (*PPARG* (A), *GLUT4* (B)), adipocyte function (*ADP2* (C), *ADIPOQ* (D), *LEP* (E)), proliferation (*PCNA* (F)) and inflammation (*IL6* (G)) in SGBS adipocytes treated with 10 nM E3 for 2, 8, and 24 hours.

**
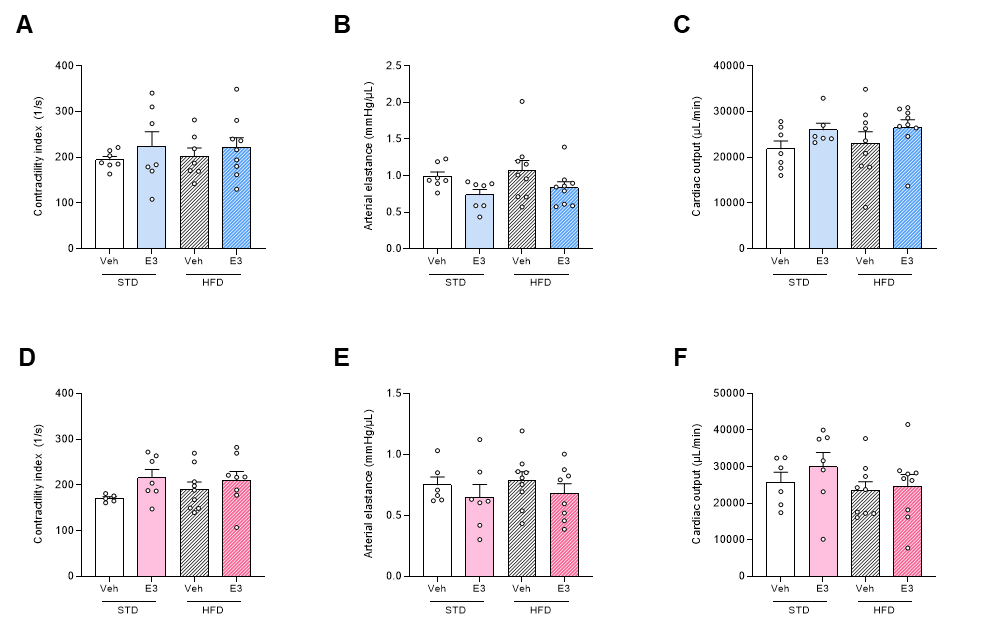
**

**Supplemental Figure S3. Effect of E3 on cardiac function parameters.** Changes in the contractility index (A, D), arterial elastance (B, E) and cardiac output (C, F) in males (A-C) and females (D-F) on standard (STD) and high-fat diet (HFD), (N=5-11 per group).

**Supplemental Figure S4. Circulating level of E3 in male mice.** Plasma concentration of E3 in male mice on standard (STD) and high-fat diet (HFD), (N=6-8 per group).

**
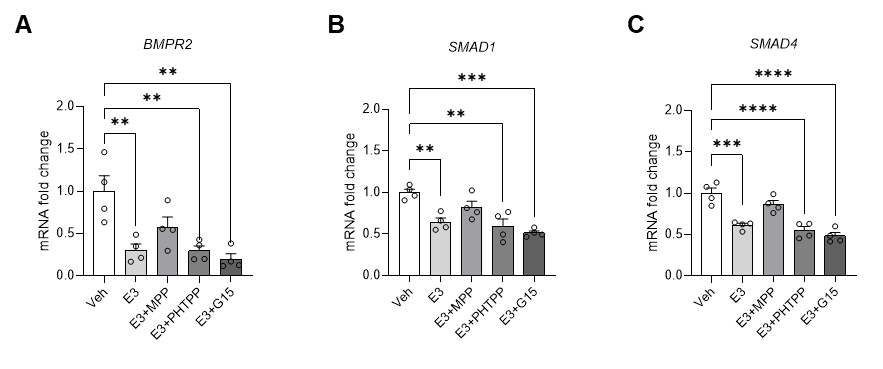
**

**Supplemental Figure S5. Estrogen receptor antagonists and BMPR signaling.** Gene expression changes in *BMPR2* (A), *SMAD1* (B), and *SMAD4* (C) in male rat PASMCs treated with E3 alone or in combination with 1 µM MPP (ERα antagonist), 1 µM PHTPP (ERB antagonist) and 1 µM G15 (GPER antagonist) for 24 hours. Data is shown as mean of four independent experiments.

**References**

1. Karen Percival ZR. Comparison of five elastin histochemical stains to identify pulmonary small vasculature. *Journal of Histotechnology* 2017;**40**:73-78.

2. Tews D, Brenner RE, Siebert R, Debatin KM, Fischer-Posovszky P, Wabitsch M. 20 Years with SGBS cells - a versatile in vitro model of human adipocyte biology. *Int J Obes (Lond)* 2022;**46**:1939-1947.

3. Denver N, Khan S, Stasinopoulos I, Church C, Homer NZ, MacLean MR, Andrew R. Derivatization enhances analysis of estrogens and their bioactive metabolites in human plasma by liquid chromatography tandem mass spectrometry. *Anal Chim Acta* 2019;**1054**:84-94.
